# Synergistic effects of inhibiting the MNK-eIF4E and PI3K/AKT/ mTOR pathways on cell migration in MDA-MB-231 cells

**DOI:** 10.18632/oncotarget.24354

**Published:** 2018-01-31

**Authors:** Ella Lineham, Graham J. Tizzard, Simon J. Coles, John Spencer, Simon J. Morley

**Affiliations:** ^1^ Department of Biochemistry, School of Life Sciences, University of Sussex, Falmer, Brighton, UK; ^2^ UK National Crystallography Service, School of Chemistry, University of Southampton, Highfield, Southampton, UK; ^3^ Department of Chemistry, School of Life Sciences, University of Sussex, Falmer, Brighton, UK

**Keywords:** cell signaling, migration, kinase, dual inhibitors

## Abstract

The study of eukaryotic initiation factor 4E (eIF4E) is a key focus in cancer research due to its role in controlling the translation of tumour-associated proteins, that drive an aggressive migratory phenotype. eIF4E is a limiting component of the eIF4F complex which is a critical determinant for the translation of mRNAs. Mitogen-activated protein kinase interacting protein kinases (MNK1/2) phosphorylate eIF4E on Ser209, promoting the expression of oncogenic proteins, whereas mTORC1 phosphorylates and de-activates the eIF4E inhibitor, 4E-BP1, to release translational repression. Here we show that inhibiting these pathways simultaneously effectively slows the rate of cell migration in breast cancer cells. However, a molecular hybridisation approach using novel, cleavable dual MNK1/2 and PI3K/mTOR inhibiting hybrid agents was less effective at slowing cell migration.

## INTRODUCTION

Metastasis of cancer cells and the formation of malignant secondary tumours is extremely problematic in the clinic, accounting for approximately 90% of human cancer deaths [1]. Cancer cells require elevated protein synthesis to invoke this invasive phenotype, which has the ability to bypass tissue barriers, intravasate into the bloodstream and seed at distal secondary sites [2]. This is particularly apparent in Triple-Negative Breast Cancer (TNBC), a rapidly spreading, primary breast cancer that has poor prognosis and limited treatment options [3].

MAPK-interacting kinases (MNKs) are serine/ threonine kinases that lie downstream of essential signaling pathways, and are commonly amplified in cancer cells [4]. Each MNK gene, *MKNK1* and *MKNK2*, produces a long and a short isoform through variation in splicing [5, 6]. The longer isoforms (MNK1a and MNK2a) contain a MAPK binding site, which is lacking in the shorter isoforms (MNK1b and MNK2b) [5, 6]. The MNKs differ in their regulation; MNK1a has low basal activity, and is activated and tightly regulated by ERK and p38 kinases in response to mitogens and stress [7, 8]. MNK2 displays high basal activity and is predominantly regulated by ERK1/2, although MNK2a is regulated by mTORC1 through at least one site in its C-terminal region [9, 10].

The eukaryotic translation initiation factor 4E (eIF4E) is the rate-limiting component of the eIF4F complex, essential for cap-dependent translation. Its availability is regulated by mTORC1, through the phosphorylation of 4E-binding protein 1 (4E-BP1). MNK1/2 phosphorylate S209 of eIF4E, promoting the expression of oncogenic proteins. Phosphorylated eIF4E (eIF4E-P) preferentially enhances the translation of a subset of mRNAs involved in cell survival. These include a number of genes implicated in metastatic and invasive behaviour, such as C-MYC, cyclin D1, PIM-1, survivin, BCL-2, vascular endothelial growth factor (VEGF), fibroblast growth factor (FGF-2) and matrix metalloproteinase-9 (MMP9) [11, 12]. This addiction to increased protein synthesis provides a therapeutic window to selectively target the translational machinery of cancer cells [13, 14].

MNK1/2 represent an attractive potential therapeutic target as they act at the convergence point of two critical signaling pathways; p38MAPK and ERK, which are often subject to up-regulation in tumour cells [4, 15–17]. MNK1/2 knockout mice exhibit total abrogation of eIF4E phosphorylation and display no developmental or reproductive defects [15, 18]. Furthermore, knock-in mice expressing a mutant form of eIF4E (S209A) are no longer phosphorylated on eIF4E by MNK1/2 and exhibit resistance to neoplastic transformation [19].

There is evidence for a compensatory feedback mechanism linking the PI3K-AKT-mTOR and the MNK-eIF4E pathways. In prostate and lung cancer, down-regulation of one pathway correlated with the activation of the other, which subsequently promoted cell growth and cancer survival [18, 20]. Dual inhibition of MNK1/2 kinase and mTORC1 suppressed cell cycle progression and blocked proliferation in both prostate and glioblastoma cell lines. This effect was increasingly pronounced when inhibitors were added in combination, indicating the importance of dual abrogation of such pathways [18, 20]. Recently published work also found that increased levels of eIF4E-P was a common feature in breast cancer patient response to chemotherapy and was associated with poor clinical outcome. Treatment with MNK1/2 inhibitors sensitised breast cancer cells to chemotherapy *in vivo* and resulted in an enhanced response to treatment [21].

The heterogeneous nature of cancer and complexity of cellular signalling means that the traditional single treatment approach is often ineffective. Inhibition of multiple targets is required to outsmart the tumour cell [13, 22, 23]. This has led to the concept of hybrid drugs, which involves the linking of two selected pharmacophores that act against different therapeutic intracellular targets simultaneously. This multi-hit approach may lead to an increase in synergy and make drug resistance less likely to occur [18].

There are no FDA approved drugs that specifically act on MNK1/2. The pyrazolo-pyrimidine compound CGP57380 exhibits low micromolar MNK1 inhibition and cercosporamide, a natural anti-fungal agent, was found to be a potent inhibitor of MNK1/2 [4, 7, 9, 11, 14, 15, 17]. However, the broad spectrum effects of cercosporamide limit its uses as an effective treatment [4, 24, 25]. Due to our interest in developing a hybrid therapy we sought to identify a MNK1/2 inhibitor with the characteristics to facilitate the development of such agents. A potent MNK1/2 inhibitor, 1, has recently been synthesised, which is relatively selective compare with current inhibitors [26].

**Figure 1:**
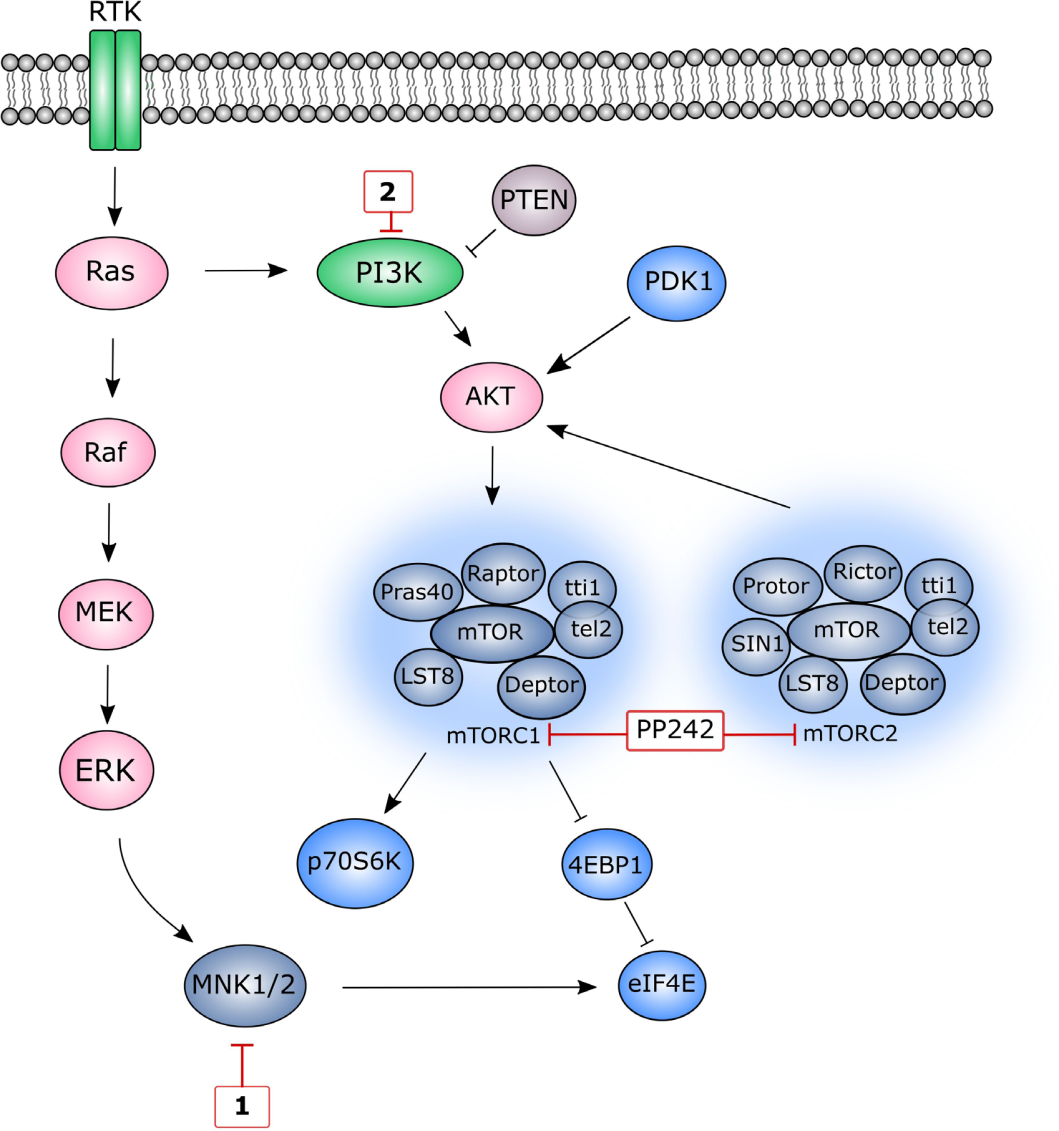
A simplified schematic representation of MNK1/ 2 and mTOR pathways Red boxes depict several inhibitors of specific pathway components used in this study. MNK1/2 lie at the convergence point of both the p38MAPK pathway and ERK pathways and upon activation, phosphorylate eIF4E. Class I PI3 kinases are activated by RTKs resulting in the conversion of PIP2 to PIP3, a secondary messenger that is essential for AKT translocation to the plasma membrane. The level of PIP3 is negatively regulated by the tumour suppressor, PTEN. AKT is partially activated by PDK1 and becomes fully activated upon phosphorylation at Ser473, a process that can be catalysed by multiple proteins. mTOR forms two distinct multiprotein complexes, mTORC1 and mTORC2. mTORC1 is activated indirectly through AKT. Activated mTORC1 stimulates protein translation by phosphorylating 4E-BP1 on several residues, releasing eIF4E allowing it to participate in translation initiation. In addition to phosphorylating other translational targets, mTORC1 also phosphorylates p70S6 kinase (p70S6K), which becomes fully activated following PDK1-mediated phosphorylation.

In this study, we examined the effect of dual inhibition of MNK-eIF4E and PI3K-AKT-mTOR pathways (Figure 1) on cell migration, cell viability and cell cycle arrest. These data led to the attempted development of dual action MNK hybrid agents, with a view to simultaneously inhibit these pathways.

## RESULTS

### The effect of dual-inhibition of both eIF4E-MNK and PI3K-AKT-mTOR pathways on downstream signalling molecules in MRC5 cells

**Figure 2:**
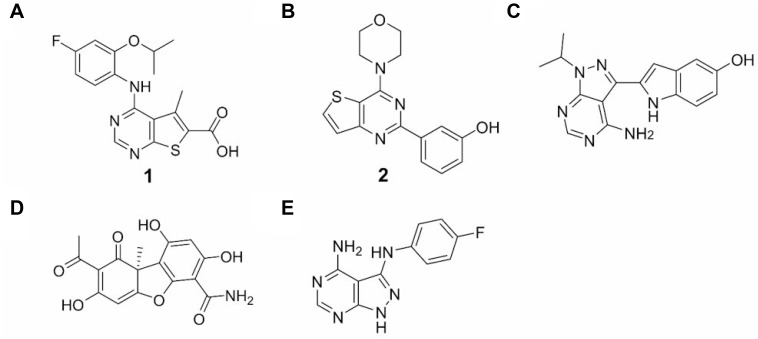
Inhibitors (**A**) Structure of 1, a MNK1/ 2 inhibitor. (**B**) Structure of 2, a PI3K p110α inhibitor. (**C**) Structure of PP242, a mTORC1/2 inhibitor. (**D**) Structure of cercosporamide, a MNK1/2 inhibitor. (**E**) Structure of CGP57380, a MNK1/2 inhibitor.

**Figure 3:**
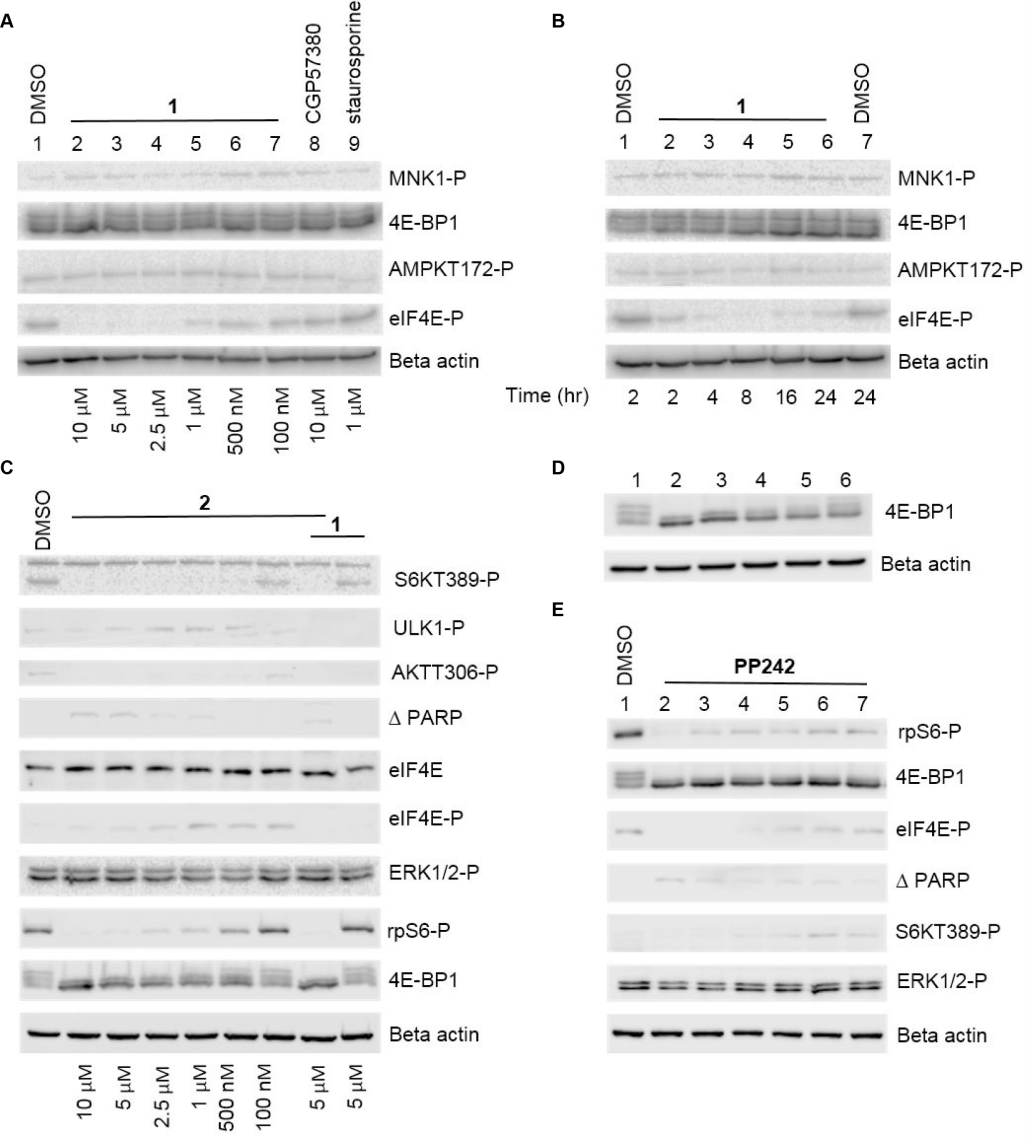
Representative Western blot analysis of both the eIF4E-MNK and PI3K-AKT-mTOR pathways in the presence of inhibitors (**A**) Western blot analysis showing the effect of different concentrations of 1 on eIF4E-P and various signalling molecules. MRC5 fibroblast cells were incubated with DMSO alone (lane 1) or with the indicated concentrations of inhibitors for 24 hours. Cellular lysates were prepared and immunoblotting was performed using 20 μg of total lysate protein, as described in the Materials and Methods. (**B**) Western blot analysis showing the effect of 1 incubation time on eIF4E-P and various signalling molecules. MRC5 fibroblast cells were incubated with DMSO alone (lane 1) or with 1 (5 μM) for the indicated times. (**C**) Western blot analysis showing the effect of different concentrations of 2 on AKT-308 phosphorylation and various signalling molecules and the effect of 2 in combination with 1. MRC5 fibroblast cells were incubated with DMSO alone (lane 1) or with the indicated concentrations of inhibitors for 24 hours. (**D**) Western blot analysis showing the effect of different concentrations of PP242 on 4E-BP1 phosphorylation. MRC5 cells were incubated with DMSO alone (lane 1) or the following final concentrations of PP242: lane 2: 5 μM, lane 3: 2.5 μM, lane 4: 1 μM, lane 5: 500 nM, lane 6: 100 nM. (**E**) Western blot showing the effect of different concentrations of 1 in combination with PP242 1 μM. Cells were incubated with DMSO alone (lane 1) or the following final concentrations of inhibitors: lane 2: 1 10 μM, PP242 1 μM, lane 3: 1 5 μM, PP242 1 μM, lane 4: 1 2.5 μM, PP242 1 μM lane 5: 1 1 μM, PP242 1 μM, lane 6: 1 500 nM, PP242 1 μM lane 7: 1 100 nM, PP242 1 μM.

MNK1/2 phosphorylate eIF4E on S209, enhancing the translation of specific mRNAs involved in cell survival and metastasis. MNK1/2 kinases are an attractive therapeutic target as they are dispensable during normal development and hence could be used to selectively kill cancer cells [26]. In our study, highly migratory MRC5 lung fibroblasts and MDA-MB-231 breast cancer cells (wildtype PI3K and BRCA1, mutated KRas (G13D) [28] and mutated P53 (missense mutation) [29]) were both used to probe the response of dual-inhibition of the eIF4E-MNK pathway and PI3K-AKT-mTOR pathways (Figure 1). Compound 1 (Figure 2), was found to be a potent MNK1/2 inhibitor in the nanomolar range in cell-free kinase assays [30]. In our hands, Compound 1 reduced the level of eIF4E phosphorylation in a concentration-and time-dependent manner, being effective at concentrations above 1 μM for 4 hours or longer (Figure 3A and 3B). In contrast, CGP57380 (Figure 3A, lane 8) and staurosporine (Figure 3A, lane 9) had no effect on eIF4E-P under these assay conditions. The inhibition of MNK1/2 did not affect the upstream activation of MNK1/2, or lead to an increase in cell stress, as observed by the constant level of AMPK T172 phosphorylation in relation to the DMSO control. Increased incubation time with 1 for 16 hours and above, resulted in partial inhibition of 4E-BP1 phosphorylation, as depicted by an increase in the level of the less-phosphorylated form of 4E-BP1 upon Western blotting (Figure 3A and 3B).

To assess the significance of simultaneous MNK1/2 and PI3K inhibition, an effective PI3K p110α inhibitor, 3-[4-(4-morpholinyl)thieno[pyrimidin-2-yl]phenol, 2, [31], was used to observe the effect on various signalling molecules (Figure 2 and Figure 3C). The efficacy of 2 as a PI3K inhibitor was measured by monitoring the inhibition of phosphorylation of AKT-T308. This phosphorylation is PI3K-dependent, resulting from the conversion of PIP2 to PIP3 and the activation of PDK1 [32]. Molecule 2 was found to inhibit AKT-T308 phosphorylation at concentrations greater than 100 nM in MRC5 cells. The observed effect of 2 on mTOR signalling was also as predicted, with mTORC1 substrates, 4E-BP1, p70S6K and consequently ribosomal protein S6 phosphorylation inhibited in a dose-dependent manner (Figure 3C). This is explained by the shared sequence similarity in the C-terminal kinase domain of both mTOR and PI3K [33]. Interestingly, 2 appeared to reduce the level of eIF4E-P at high concentrations, suggesting an overlapping function in the eIF4E-MNK pathway. The phosphorylation of eIF4E was abolished when 2 was used in combination with 1.

When monitored by Western blotting, the level of eIF4E protein remained constant throughout the drug-treated cells. A low level of cleaved PARP was observed when 2 was used at high concentrations alone or in combination with 1 (Figure 3C), indicating apoptosis at elevated final concentrations.

In addition to targeting the PI3K pathway at the level of PI3K, we used the pyrimidine derivative, PP242 (torkinib), to inhibit this pathway. PP242 has an IC_50_ of 8 nM in cell-free assays and selectively targets both mTORC1 and mTORC2 complexes over PI3K isoforms [34] (Figure 1). Consistent with published data [34], PP242 at a 1 μM final concentration inhibited the phosphorylation of 4E-BP1 (Figure 3D and 3E) as well as other targets downstream of mTORC1, including p70S6K and ribosomal protein S6 (data not shown). There was a low level of cell death in cells exposed to the 1/ PP242 combination, with more cleaved PARP observed at the highest concentration of 1 (10 μM; lane 2). Incubation of cells with PP242 at 1 μM concentration in combination with various concentrations of 1 did not affect the efficacy of the latter on eIF4E-P. The level of ERK-P appeared to be unaffected under all of the conditions tested.

### Inhibition of MNK1/2 and the PI3K-AKT-mTOR pathway slows the rate of migration in MDA-MB-231 cells

**Figure 4:**
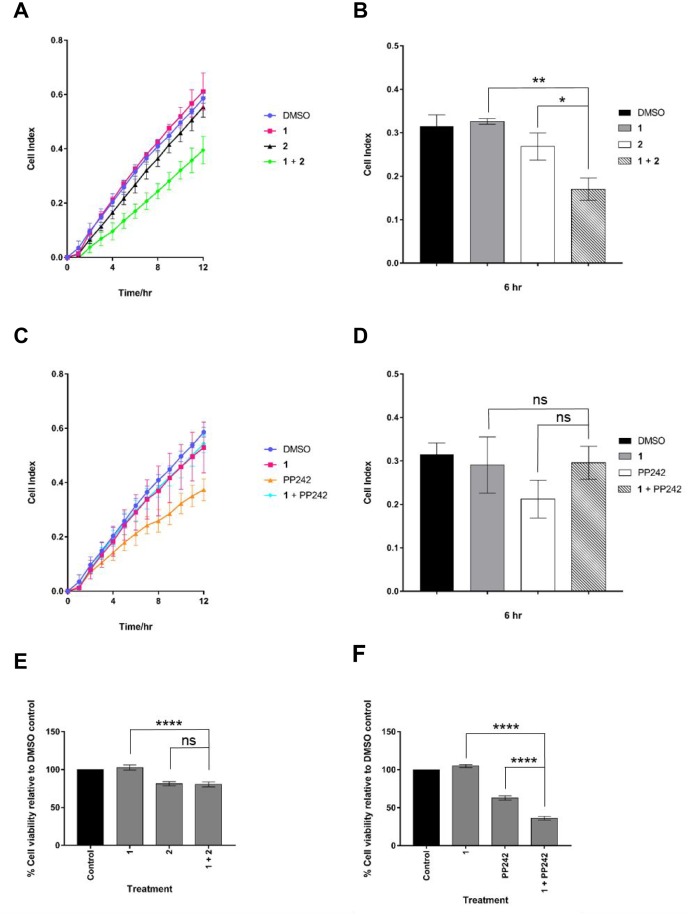
Cell viability and kinetics of migration in drug-treated MDA-MB-231 cells Cell migration in real time was analysed by the xCELLigence RTCA. (**A**) and (**C**) show the cell indexes over 12 hours for each drug treatment at 1 μM. (**B**) and (**D**), cell migration analysis at 6 hr. (**E**) and (**F**) Viability was assessed by Cell Titer Blue assay. MDA-MB-231 cells were treated for 72 hours with indicated drugs at 1 μM. Data are mean ± S.D, *n* = 3 (^*^p ≤ 0.05; ^**^p ≤ 0.01; ^***^p ≤ 0.001 and ^****^p ≤ 0.0001).

The phosphorylation of eIF4E correlates with an increase in levels of mesenchymal markers such as N-cadherin, fibronectin and vimentin, along with the acquisition of invasive properties [35]. Disrupting cellular migration is a promising therapeutic option for the treatment of cancer [36]. Here, we assessed the rate of migration of a breast cancer cell line using real-time monitoring of cell migration. MDA-MB-231 cells were analysed in the presence of various inhibitors alone or in combination as they moved towards a chemo-attractant. Cell migration kinetics were recorded on a RTCA DP instrument for 12 hours. As shown in Figure 4A and 4B, when cells were treated with a combination of 1 and 2 (both at 1 μM final concentration), a substantial reduction in cell migration was observed relative to the DMSO control. In comparison, when used as single agents, both 1 and 2 had negligible effects on cell migration. Cell viability data demonstrated that the MNK1/2 inhibitor, 1, exhibited minimal cytotoxicity at 1 μM in MDA-MB-231 cells (Figure 4E and 4F). The PI3K inhibitor, 2, reduced cell viability to 75%, when used either as a single agent or in combination with 1.

The synergistic effect of MNK1/2 and PI3K inhibition on cell migration led to the investigation of inhibition of both MNK1/2 and mTORC1/2, using PP242. The latter, impaired cell migration to the greatest extent (Figure 4C), although this could in part be explained by a reduction in cell viability to 60% seen with this compound (Figure 4F). A combination of 1 with PP242 appeared to rescue the effect of PP242 used alone, with the cell index returning to the level of the DMSO control (Figure 4C). One possible explanation for this reflects a recent publication demonstrating that MNK forms a complex with mTORC1, promoting mTORC1 association with TELO2 (Phosphatidyl Inositol 3ʹ Kinase-related Kinase (PIKK) stabiliser), which facilitates efficient mTORC1/substrate binding [37].

The dual inhibition of MNK1/2 and mTORC1/2 significantly reduced cell viability to 35% on control levels (Figure 4F), an observation that has been reproduced in several cancer cell lines [18, 20]. The rescue effect seen when a combination of MNK1/2 and mTORC1/2 inhibition was used in the cell migration assay suggests only a selected pool of surviving cells were scored in this assay.

### The combination of MNK1/2 and mTORC1/2 inhibition induces G_1_ cell cycle arrest in MDA-MB-231 cells

**Figure 5:**
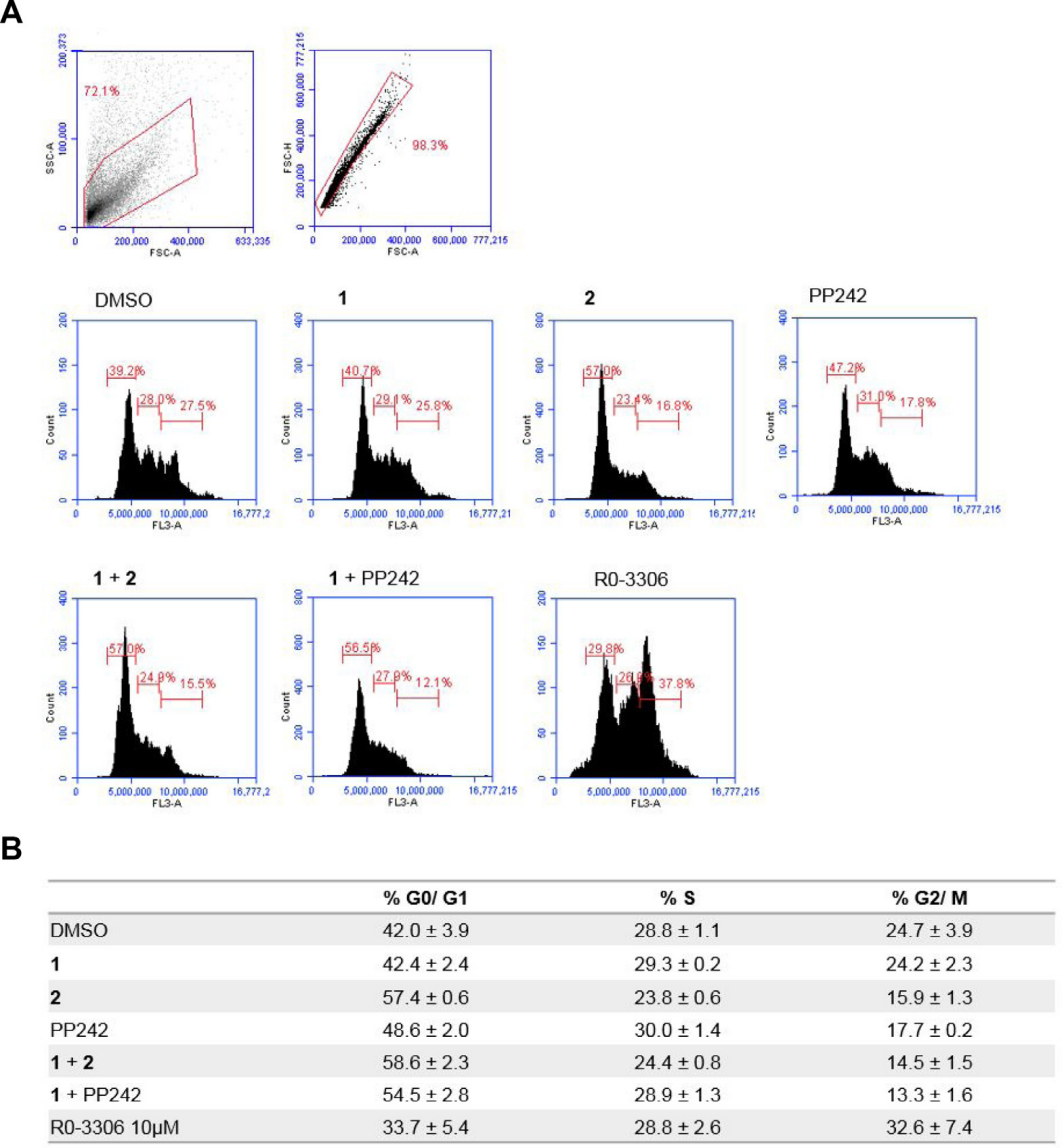
Flow cytometry analysis of the effect of MNKI-19, 191.3 and PP241 both as single agents and in combination (5 μM) on cell cycle progression (**A**) Representative plots for MDA-MB-231 cells treated for 24 hr with indicated inhibitors at 5 μM unless otherwise stated in the data table. (**B**) Data are mean ± S.D, *n* = 2.

Next, we investigated whether a combination of MNK1/2 and PI3K or mTORC1/2 inhibition was associated with cell cycle arrest in proliferating MDA-MB-231 cells over a period of 24 hours. The CDK1 inhibitor, R0-3306, was used as a control for the arrest of cells at the G_2_/ M phase border [38]. As demonstrated in Figure 5, each phase of the cell cycle showed a normal distribution in the DMSO control cells. The MNK1/2 inhibitor, 1, had no effect on cell cycle distribution, whereas both mTORC1/2 and PI3K inhibition, (PP242 and 2, respectively) increased the number of cells in the G_1_ phase of the cell cycle. However, following exposure to a combination of 1 and PP242 at 5 μM, the number of cells corresponding to the G_1_ phase was increased, while the numbers of cells in the S and G_2_/M phases were decreased. This combination of inhibitors had a synergistic effect on G_1_ cell cycle arrest.

### Chemistry

**Figure 6:**
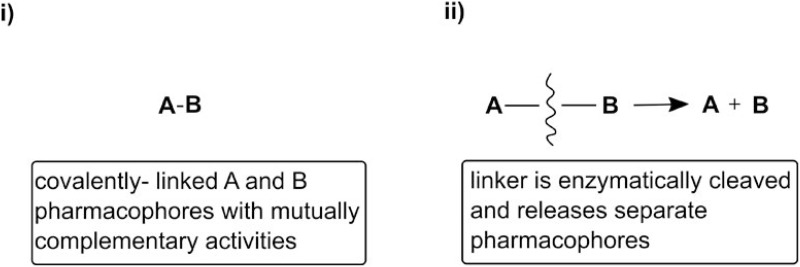
“Permanent” vs. cleavable hybrid approach

Based upon the observations above and literature precedence, we postulated that a compound containing both MNK1/2 and PI3K/mTOR inhibitory activities might be very effective against cellular migration. A further aim of this work was to synthesise novel hybrid molecules based upon 1 and either PP242 or 2 used in this study. The desired outcome was the development of a novel, single molecular framework incorporating both moieties (Supplementary Experimentals 1, 2 and Supplementary Figure 1). The hybrid concept covers either permanent or prodrug, cleavable dual-action molecules, i) or ii) (Figure 6).

**Figure 7:**
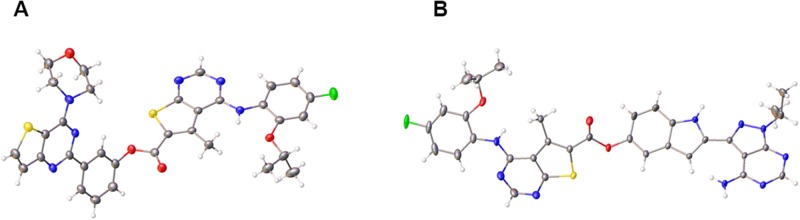
Crystal structures of hybrid agents (**A**) Compound 6 (CCDC 1813013), (**B**) Compound 7 (CCDC 1813012).

The “type ii)” hybrid compounds (6 and 7) were prepared as outlined in Supplementary Scheme 1. Compound 6 was prepared via a simple one-pot synthesis as detailed in the ESI. Compound 7 was prepared by initially converting the carboxylic acid functionality of the starting material, 1 [30] into an acid chloride. This intermediate then underwent an esterification reaction with PP242, leading to the formation of 7 in the presence of base. The final products were fully characterised by HRMS and ^1^H NMR (Supplementary Experimentals 1, 2 and Supplementary Figure 1). Single crystals of both 6 and 7 were isolated from a chloroform solution and the resulting X-ray analysis is depicted in Figure 7, which showed the expected connectivity i.e. the ester bonds in the products [39].

### *In vitro* analysis of hybrid agents in MRC5 cells

**Figure 8:**
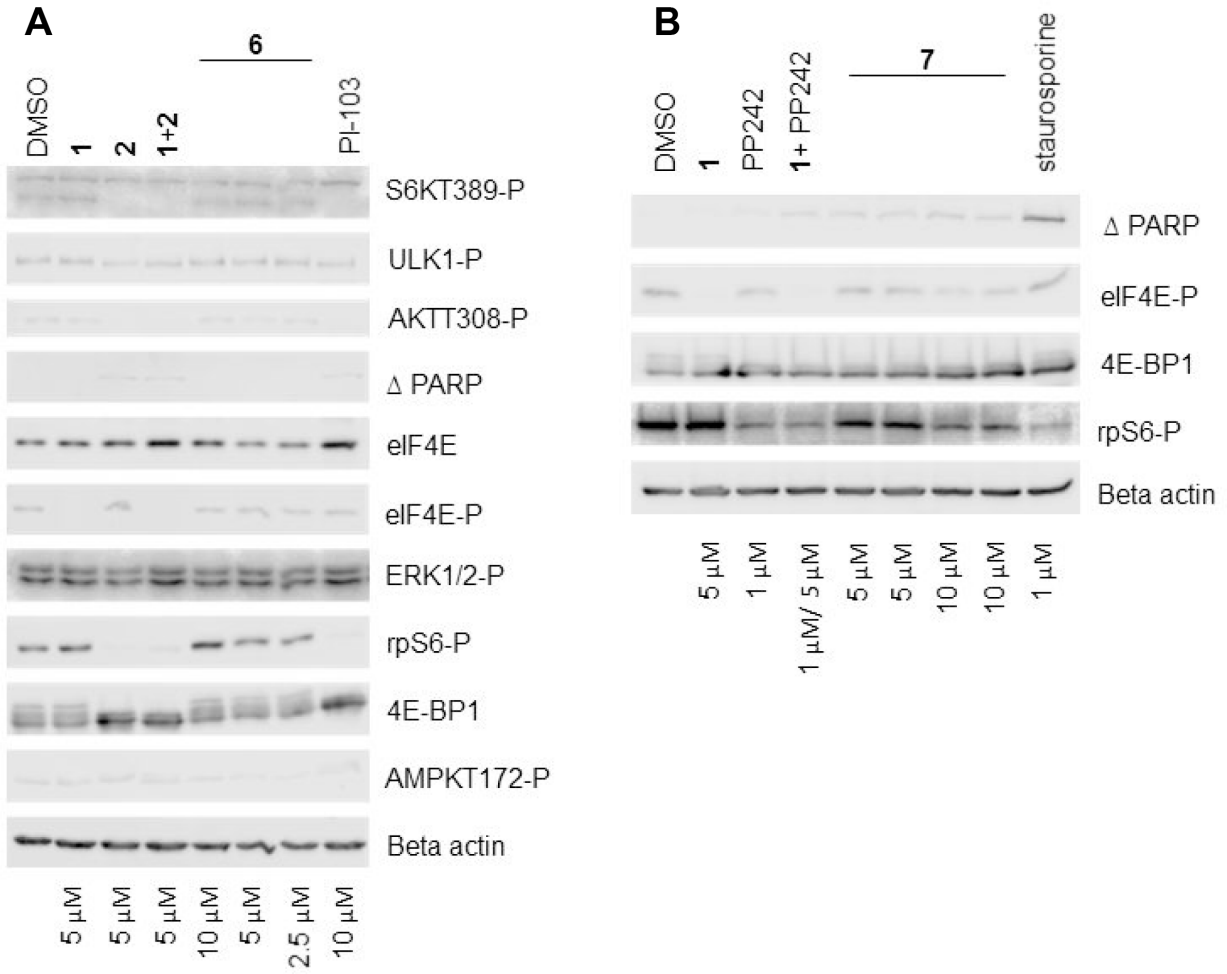
Characterisation of hybrid agents (**A**) and (**B**) Western blot analysis showing the effect of different concentrations of compounds 6 and 7 respectively on various signalling molecules. MRC5 fibroblast cells were incubated with DMSO alone or with the indicated concentrations of inhibitors for 24 hours. Cellular lysates were prepared and immunoblotting was performed using 20 μg of total lysate protein, as described in the Materials and Methods.

Compounds 6 and 7 were screened for activity in MRC5 cells by Western blotting (Figure 8A and 8B) and compared with known kinase inhibitors, PI-103 and staurosporine. Notably, both 6 and 7 were difficult to solubilise in DMSO and this was reflected in the Western blot analysis. Compound 6 failed to show any noticeable effect on either PI3K or MNK1/2 signalling output relative to the controls, represented by little change in AKT-T308-P or eIF4E-P levels, respectively. mTORC1 read-outs, 4E-BP1, S6K T389-P and phospho-ribosomal S6 protein also indicated a lack of inhibition of mTORC1 signalling. In addition, ULK-1-P and AMPK-T172-P were unaffected by the inhibitors used, indicating that cells had not activated a general stress response. When compared to the solvent control, compound 7 slightly reduced the level of eIF4E-P when tested at the highest concentration (10 μM). mTORC1 inhibition was observed at the highest concentration, reflected by the collapse of the broad 4E-BP1 signal into a condensed band and a reduction in ribosomal protein S6 phosphorylation. There was a low level of cleaved PARP in cells treated with the 1:1 mixture of 1/ PP242 and in cells treated with compound 7, suggesting that this combination in both forms contributed to cell death. The poor solubility of 6 and 7 can be explained in part by their physiochemical properties, including high molecular high weight (>500), TPSA (>140 A^2^), log P (>5) (Supplementary Table 1, ESI).

## DISCUSSION

Using MDA-MB-231 cells, we have determined that a synergistic combination of MNK1/2 and PI3K inhibitors slowed the rate of cell migration. Additionally, a combination of MNK1/2 and mTORC1/2 inhibitors resulted in cell cycle arrest and a decrease in cell viability to a greater extent in comparison to when the inhibitors were used as single agents. This led to the attempted development of novel hybrid entities, encapsulating MNK1/2 and PI3K/ mTOR inhibitory activities. Although molecular hybridisation approaches hold significant promise, they currently face considerable challenges as therapeutic modalities [40–48]. For example, both 6 and 7 are undoubtedly poorly soluble due to their high molecular weight and lipophilicity. However, simultaneous MNK1/2 and PI3K/mTOR inhibition warrants further investigation as a therapeutic option for treating aggressive migratory cancers [49].

## MATERIALS AND METHODS

### Cell culture and cell viability assays

MRC5 human male foetal lung fibroblasts, SV40 transformed, and MDA-MB-231, human Caucasian female breast adenocarcinoma cells were sourced from the European Collection of Cell Cultures (ECACC) and maintained in Minimal Essential Medium with Glutamax and Earl's salts (MEM, Gibco), supplemented with 10% (v/v) foetal bovine serum (FBS, Pan Biotech) at 37° C in a humidified atmosphere with 5% CO_2_. Cell passage was performed when cells were 70–80% confluent. Cells were first washed with Dubecco's Phosphate Buffered Saline without calcium chloride or magnesium chloride (DPBS, Sigma Aldrich) prior to the addition of cell dissociation agent, TripLE Select using 1 mLcm^−2^ (ThermoFisher Scientific). Cell viability was measured with the CellTiter-Blue reagent (Promega) per the manufacturer's instructions. Cells were plated in clear-bottomed 96-well plates at a density of 5000 cells per well. The inhibitors were added the following day, and cell viability was measured 24 hours later using the Synergy HT Multi-Detection Reader (BioTek). Relative cell viability at a given inhibitor concentration was determined by comparing the fluorescence to that of DMSO treated cells.

### Cell migration assay

The Oris Universal Cell Migration Assembly kit was purchased from AMS Biotechnology (Europe) Ltd and the assay was performed according to the manufacturer's instructions. Briefly, a single cell suspension (5 × 10^4^ cells/ well/ 100 μL) was loaded into stopper-loaded wells in a 96-well plate. Cells were incubated in a humidified chamber (37° C, 5% CO_2_) for 4 hours to permit cell attachment. To start cell migration, the stoppers were removed, cells were washed with sterile PBS and fresh complete medium was added. Images were taken at various indicated time points using an Optika XDS-2 light microscope, (4x objective lens). Data were analyzed with ImageJ software (National Institute of Health, Bethesda, MA, USA). Using the xCELLigence DP device from Roche Diagnostics real-time measurements of cell migration on MRC5 cells were performed. Cells were seeded at 30,000 per well in CIM-Plates 16 (Roche Diagnostics) in serum-free medium in the presence or absence of inhibitors. Full growth medium was used as a chemo-attractant in the lower chamber. As cells pass through the 8 μm pores towards the chemo-attractant they adhere to the underside of the filter, embedded with a gold micro-electrode. This produces an electrical impedance signal, which correlates with the number of migrating cells. Cell index is an arbitrary unit based upon the measured cell-electrode impedance derived by the software using the following calculation as described in reference [27].

### Immunoblotting

Cellular lysates were prepared using lysis buffer (20 mM MOPS pH7.4, 100 mM KCl, 1 mM DTT, 1 mM EDTA, 2 mM benzamidine, 25 mM NaF, 5 μg/mL leupeptin, 10 mM chymostatin, 1 μM microcystin LR, 1 X EDTA-free protease inhibitor cocktail (Roche)). The concentration of lysate protein was determined by Bradford assay (Bio-Rad). Immunoblotting was performed using the Mini-PROTEAN Tetra Cell System (Bio-Rad) with 20 μg of lysate protein. The primary antibodies used were β-actin (Abcam), cleaved PARP (Cell Signalling Technologies), AMPK(phospho-T172; Cell Signalling Technologies), eIF4E-(phospho-S209; Abcam), S6K(phospho-T389; Cell Signalling Technologies), Phospho-p44/42 MAPK (Erk1/2) (Thr202/Tyr204, Cell Signalling Technologies), 4E-BP1 (Cell Signalling Technologies), p38-MAPK (Thr180/Tyr182, Cell Signalling Technologies), Phospho-Mnk1 (T197/202; Cell Signalling Technologies), Phospho-S6 Ribosomal Protein (S240/244, Cell Signalling Technologies), eIF4E (Cell Signalling Technologies), Phospho-Akt (T308, Abcam), Phospho-ULK1 (S555, Cell Signalling Technologies) and Anti-LC3B (Sigma).

### Flow cytometry

Cells were seeded at 1 × 10^5^ in 12-well plates and allowed to adhere overnight. The cells were treated with the indicated concentrations of inhibitors for 24 hours. Samples were collected and fixed in 70% ethanol for 1 hour. For cell cycle analysis, fixed cells were treated with 10 μg/ml RNAse A for 45 minutes before the addition of 50 μg/ml propidium iodide for 15 minutes and then analysed by FACs using the Accuri C6 Flow Cytometer.

### Chemical synthesis general procedures

All reactions were carried out in air using commercial grade starting materials, solvents, and reagents. The progress of all reactions was monitored by thin layer chromatography (TLC) using commercially available glass silica gel plates (60 Å, F254). The mobile phase was generally a solvent mixture, and the visualization was undertaken using UV light. All NMR spectra were measured on a Varian NMR 500 spectrometer at 500 MHz (^1^H). Chemical shifts are quoted in parts per million (ppm; % relative to a residual solvent peak for ^1^H). Chromatographic purifications were undertaken using an ISCO purification unit, Combi Flash RF 75 PSI, using Biotage silica gel columns. LC-MS purity analyses were undertaken using a 5 μm C18 110 Å column. The synthesis of the hybrid agents is detailed in the ESI.

## SUPPLEMENTARY MATERIALS FIGURES


