# Supplementary material for: Synergistic effects of inhibiting the MNK-eIF4E and PI3K/AKT/ mTOR pathways on cell migration in MDA-MB-231 cells
Source: Oncotarget. 2018 Jan 31;9(18):14148–59. doi: 10.18632/oncotarget.24354 (PMC5865660; doi:10.18632/oncotarget.24354)
Supplement: Supplementary file 1 [file oncotarget-09-14148-s001.pdf]

Synergistic effects of inhibiting the MNK-eIF4E and PI3K/AKT/mTOR pathways on cell migration in MDA-MB-231 cells

SUPPLEMENTARY MATERIALS

A

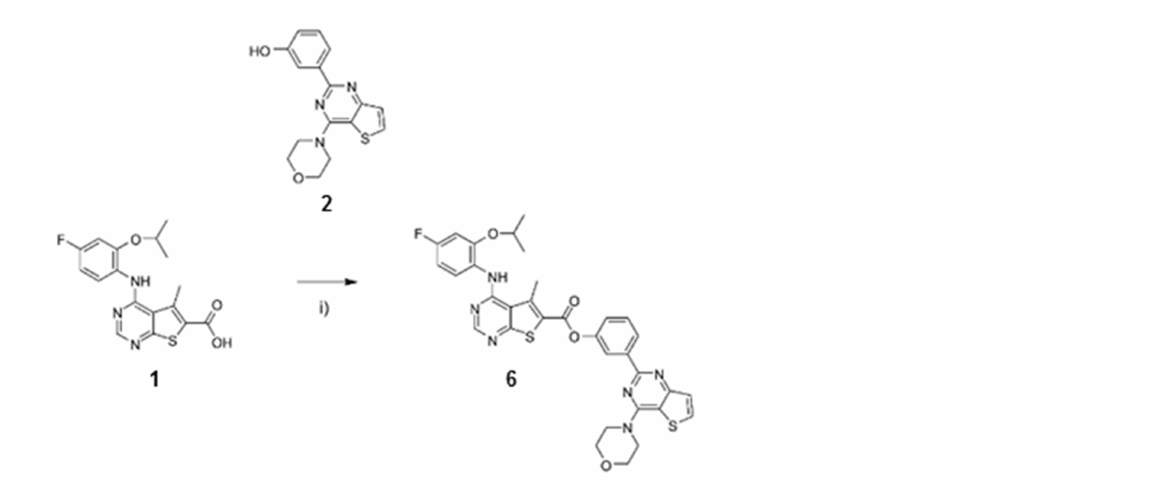

B

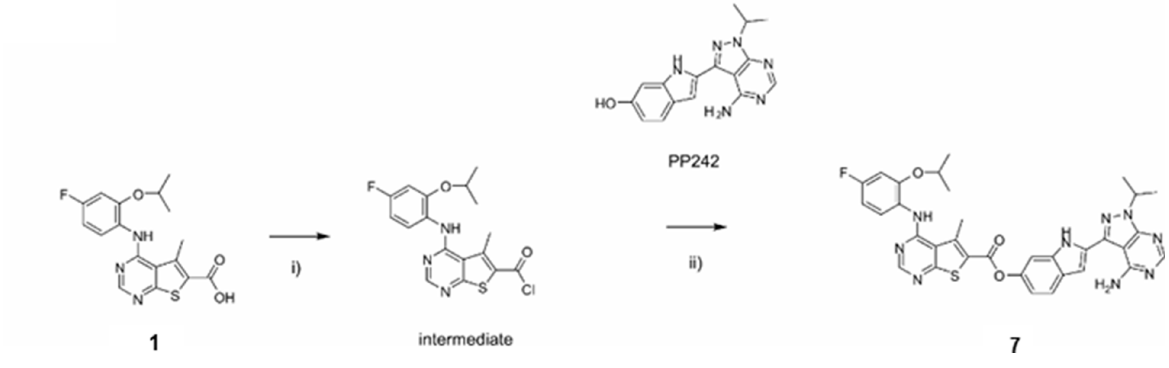

Supplementary Scheme 1: Synthesis of ester-linked, cleavable hybrid molecules. Reagents and conditions: (**A**) i) DMF, Et_3N, EDC hydrochloride, HOBt, DMAP, RT overnight (**B**) i) $SOCl_2$, toluene, reflux 2 h ii) Et_3N, CH_2Cl_2, RT overnight.

Supplementary Experimental 1: Experimental procedures for the synthesis of hybrid compound 6: 3-[4-(morpholin-4-yl)thieno[3,2-d]pyrimidin-2-yl]phenyl 4-{[4-fluoro-2-(propan-2-yloxy)phenyl]amino}-5-methylthieno[2,3-d]pyrimidine-6-carboxylate

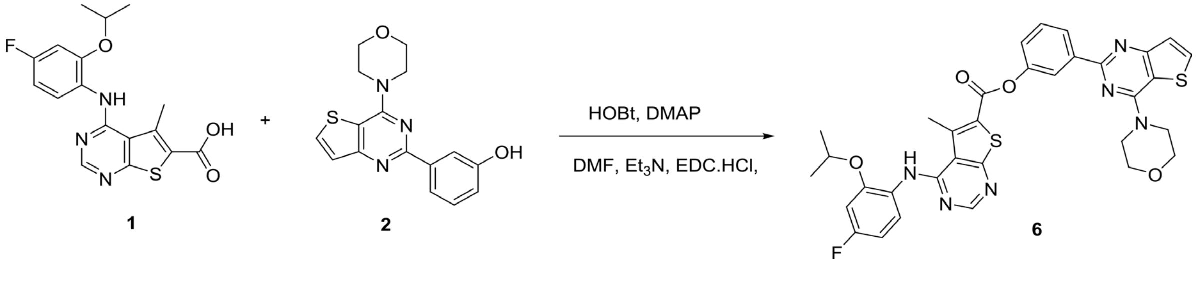

To 4-((4-fluoro-2-isopropoxyphenyl)amino)-5-methylthieno[2,3-d]pyrimidine-6-carboxylic acid, **1**, (40 mg, 0.11 mmol) in DMF (2 mL) was added 1-ethyl-3-(3-dimethylaminopropyl)carbodiimide hydrochloride (EDC.HCl) (20 mg, 0.1 mmol), 1-hydroxybenzotriazole hydrate (HOBT) (16 mg, 0.1 mmol), 3-[4-(4-morpholinyl)thieno[pyrimidin-2-yl]phenol (33 mg, 0.1 mmol), triethylamine (100 µL, 0.67 mmol) and 4-dimethylaminopyridine (catalytic). The reaction mixture was stirred at RT overnight. The reaction mixture was diluted with ethyl acetate (10 mL) and washed twice with brine and once with sodium carbonate (sat). The organic layer was separated, dried ($MgSO_4$), filtered and concentrated *in vacuo*. The residue was purified by ISCO Combiflash as above to afford an off-white solid (16 mg, 24%). Due to the low yielding reaction, no ^{13}C NMR spectrum was recorded. ^{1}H NMR ($CDCl_3$) 1.45 (6H, d, J = 6 Hz), 3.23 (3H, s), 3.90–3.93 (4H, m), 4.07–4.11 (4H, m), 4.66 (1H, sept, J = 6 Hz), 6.71 (2H, dd, J = 10, 3 Hz), 7.34–7.36 (1H, m), 7.53–7.57 (2H, m), 7.77 (1H, d, J = 5.5 Hz), 8.33 (1H, dd, J = 2.5, 1.5 Hz), 8.44–8.47 (2H, m), 8.68 (1H, s), 8.83 (1H, dd, J = 9, 6 Hz). HRMS: $C_{33}H_{29}FN_6O_4S_2$ H+; Calcd 667.1748; Found 667.1728. Elemental anal (recrystallized from CH_2Cl_2). Calcd, for $C_{33}H_{29}FN_6O_4S_2$. 0.75 CH_2Cl_2 C, 56.27; H, 4.27; N, 11.67; Fnd, C, 56.79 ; H, 4.42; N, 11.63.

Compound 6

^{1}H NMR ($CDCl_3$) (acetone signal at 2.19)

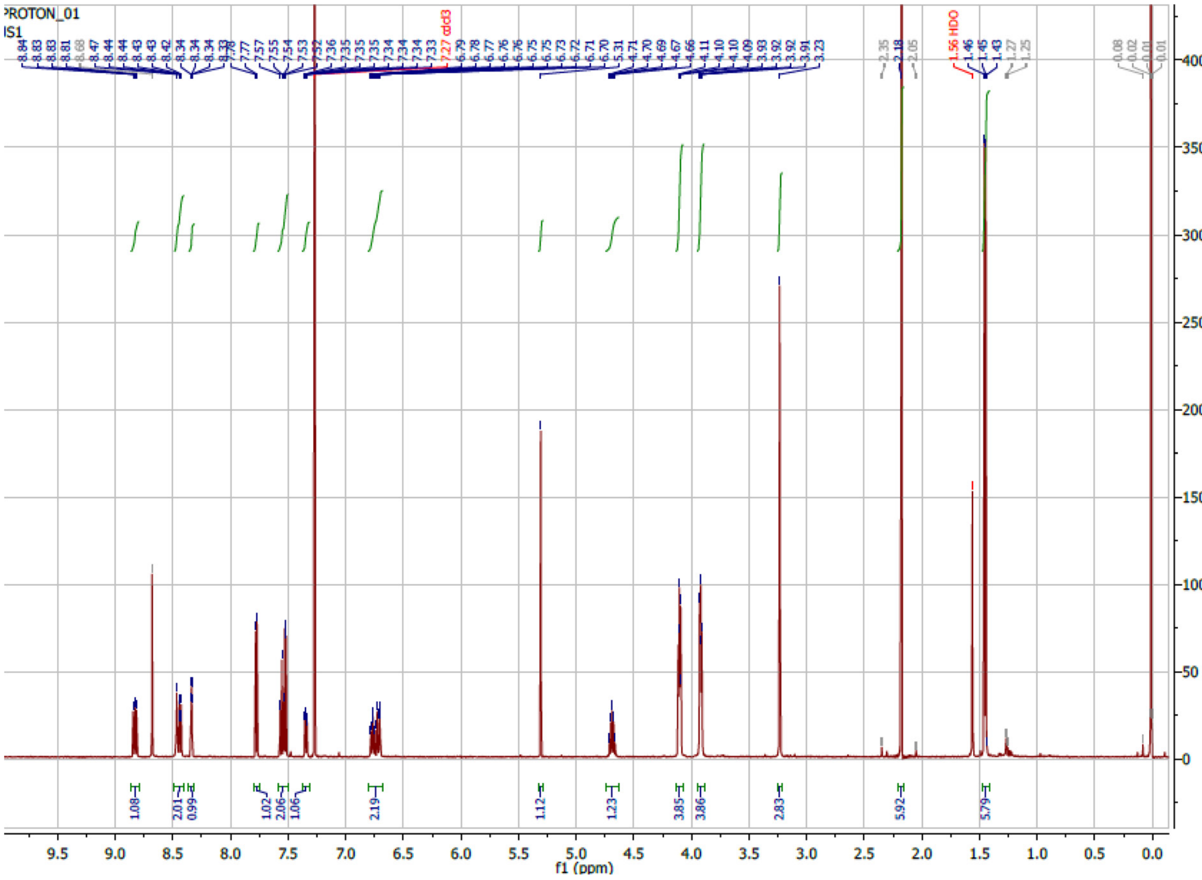

CHN analysis:

SAMPLE	%C	%H	%N
mnkpi3K	56.79 56.85	4.42 4.50	11.63 11.58

--
Stephen Boyer
Science Centre
London Metropolitan University
29 Hornsey Road
London N7 7DD

Generic Display Report

Analysis Info

Analysis Name	D:\Data\Alinanopos\JOHN_7980_A_000001.d	Acquisition Date	21/01/2017 12:20:06
Method	pos20090608esi	Operator	Administrator
Sample Name	POS ESI ELLA MOTR	Instrument	apex-III
Comment			

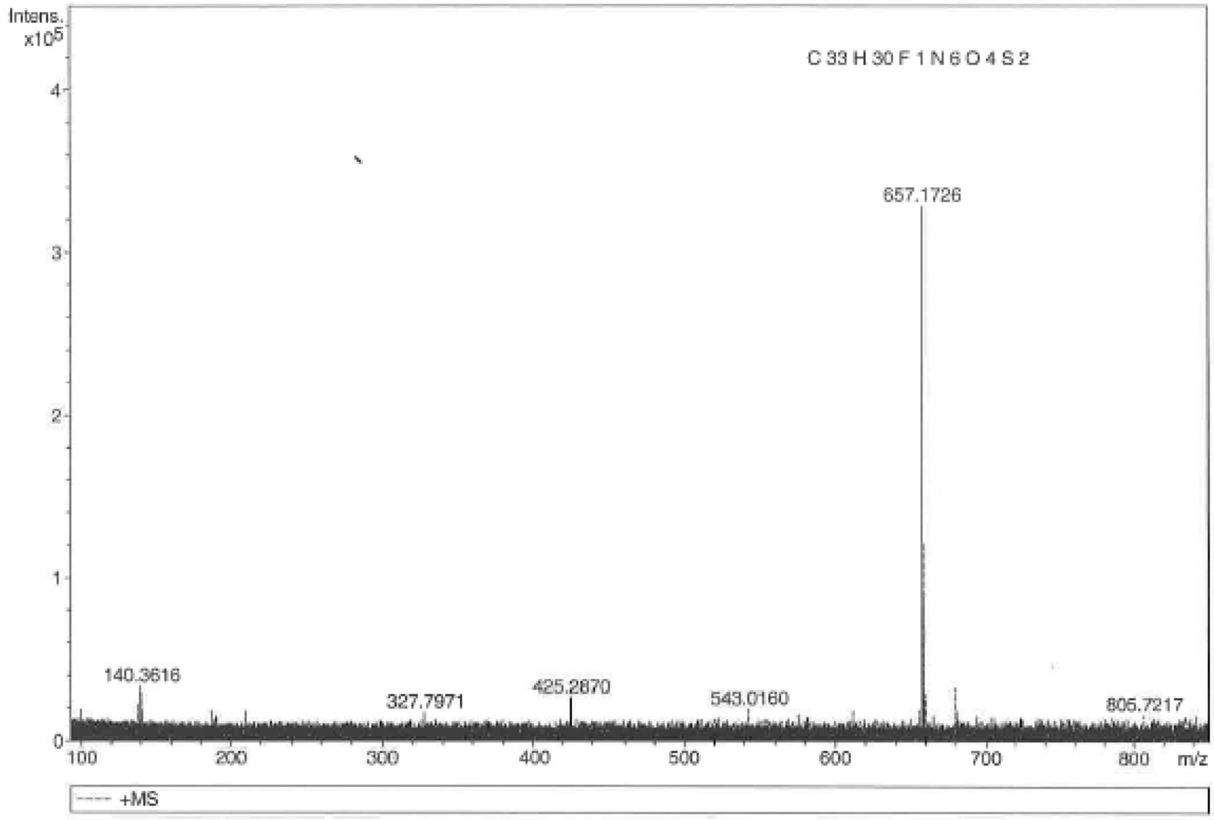

Sum Formula	Sigma	m/z	Err [ppm]	Mean Err [ppm]	Err [mDa]	rdb	N Rule	e⁻
C 33 H 30 F 1 N 6 O 4 S 2	0.047	657.1748	3.37	3.27	2.15	21.50	ok	even

Supplementary Experimental 2: Experimental procedures for synthesis of hybrid compound 7: 2-[4-amino-1-(propan-2-yl)-1H-pyrazolo[3,4-d]pyrimidin-3-yl]-1H-indol-6-yl 4-{[4-fluoro-2-(propan-2-yloxy)phenyl]amino}-5-methylthieno[2,3-d] pyrimidine-6-carboxylate

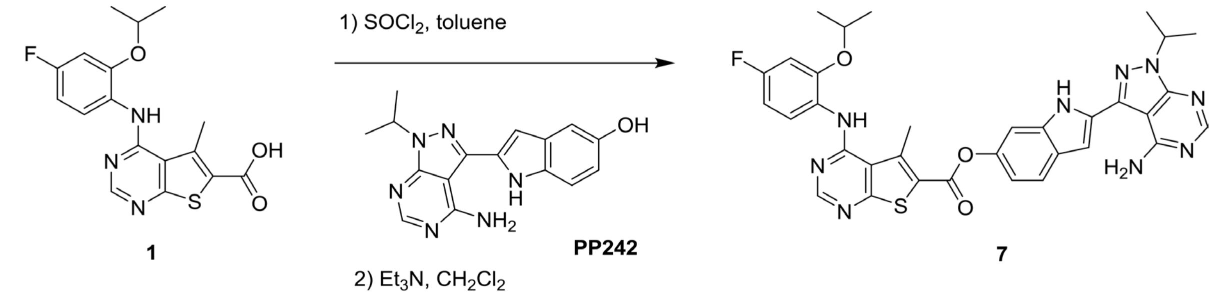

To compound **1**, 4-((4-fluoro-2-isopropoxyphenyl)amino)-5-methylthieno[2,3-d]pyrimidine-6-carboxylic acid, (39 mg, 0.11 mmol) in toluene (8 mL) was added thionyl chloride (15 µl, 0.2 mmol), one drop of dimethylformamide (DMF), and the reaction mixture was refluxed for two hours. After cooling, volatiles were removed in vacuo. The residue was re-dissolved in dichloromethane (2 mL) and reconcentrated then redissolved in dichloromethane (2 mL). To the reaction mixture was added **PP242,** 2-[4-amino-1-(propan-2-yl)-1H-pyrazolo[3,4-d]pyrimidin-3-yl]-1H-indol-5-ol, (35 mg, 0.11 mmol) and excess triethylamine (95 µl) in dichloromethane (2 mL) and the reaction mixture was stirred at RT overnight. The reaction mixture was extracted with dichloromethane (10 mL) and washed (sat. $NaHCO_3$ solution, 10 mL). The organic extracts were separated, dried ($MgSO_4$) then filtered and concentrated *in vacuo*. Pure product was obtained by chromatography on an ISCO Combiflash from dichloromethane (neat) to EtOAc/dichloromethane (9:1) gradient. A red crystalline solid (10 mg, 14 % yield) was obtained from a $CDCl_3$ solution, one crystal of which was studied in the solid state. Due to the low yielding reaction, no ^{13}C NMR spectrum was recorded. ^{1}H NMR ($CDCl_3$) 1.27 (6H, d, J = 6 Hz), 1.44 (6H,d, J = 5 Hz), 3.22 (3H, s), 4.68 (1H, sept, J = 6 Hz), 5.21 (1H, sept, J = 6 Hz), 6.04 (2H, br s), 6.73 (2H, dd, J = 10, 3 Hz), 6.90 (1H, s), 7.13 (1H, m), 7.48 (2H, d, J = 2 Hz), 8.42 (2H, d, J = 2 Hz), 8.67 (1H, s), 8.81 (1H, dd, J = 9, 6 Hz), 9.30 (1H, s). HPLC purity (>96 %). HRMS: $C_{33}H_{29}FN_6O_4S_2$. H^+ calcd; 652.2249; found 652.2256.

<u>**Compound 7**</u>

^{1}H NMR ($CDCl_3$)

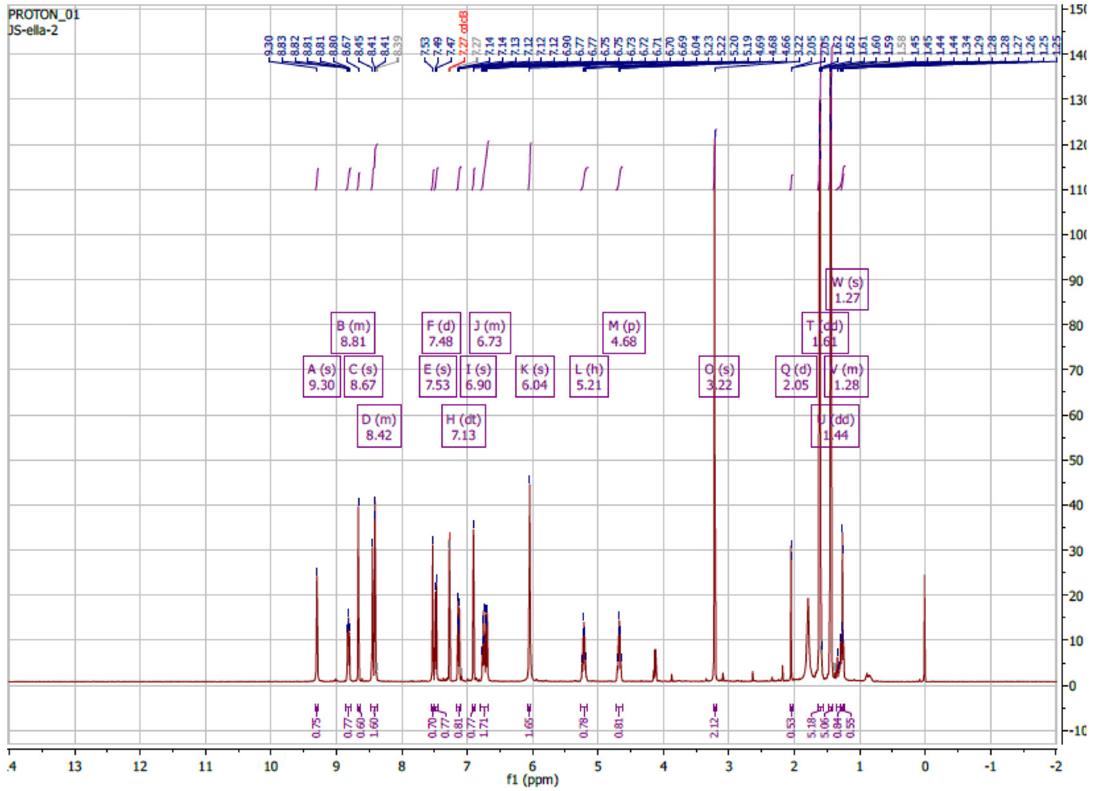

Generic Display Report

Analysis Info

Analysis Name	D:\Data\Alinanopos\JOHN_7535_000001.d	Acquisition Date	22/08/2016 15:54:54
Method	pos20090608esi		
Sample Name	POS ESI POS JS ELLA HYBRID	Operator	Administrator
Comment		Instrument	apex-III

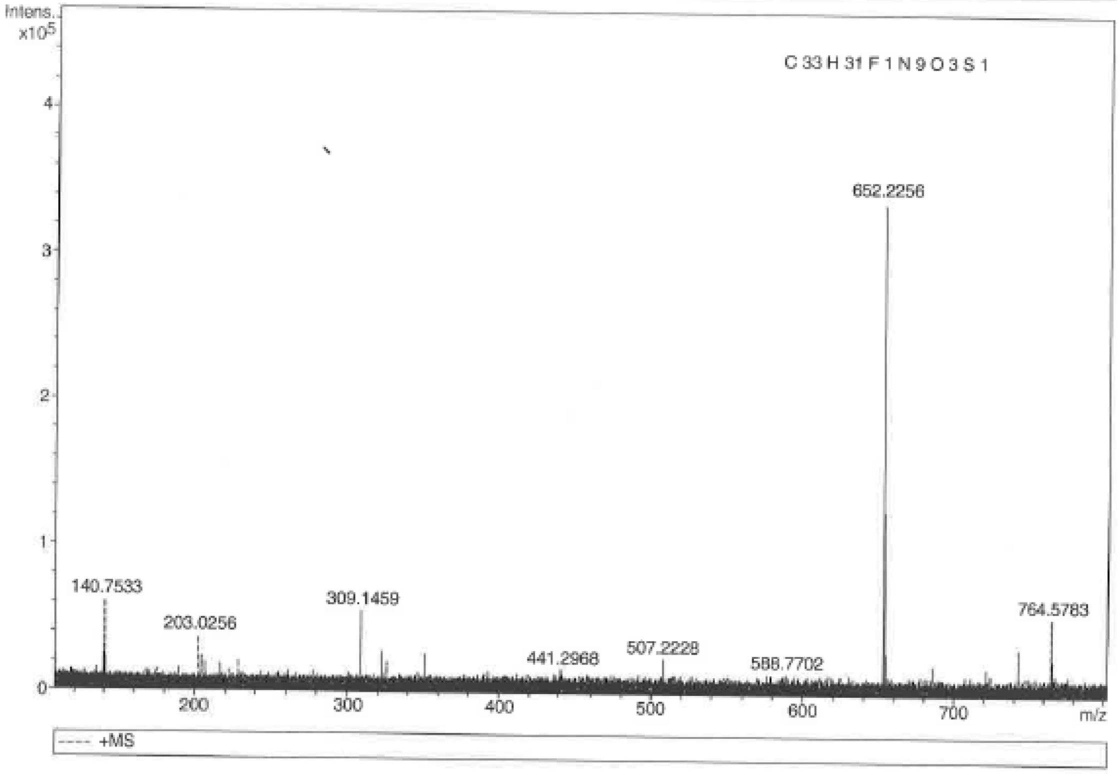

Sum Formula	Sigma	m/z	Err [ppm]	Mean Err [ppm]	Err [mDa]	rdb	N Rule	e⁻
C 33 H 31 F 1 N 9 O 3 S 1	0.032	652.2249	-1.12	-1.97	-1.28	22.50	ok	even

% purity LC-MS

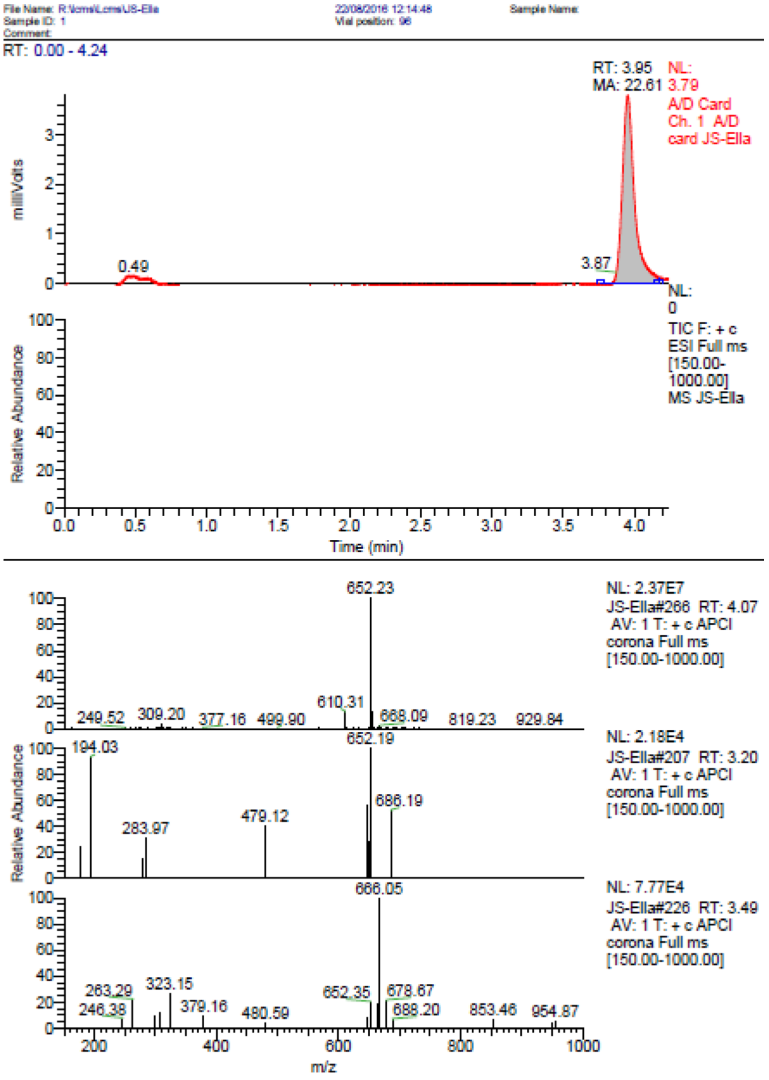
File Name: R:\lcms\Lcms\JS-Ella
Sample ID: 1
Comment:
22/08/2016 12:14:48
Vial position: 96
Sample Name:
RT: 0.00 - 4.24
RT: 3.95
MA: 22.61
NL: 3.79
A/D Card Ch. 1 A/D card JS-Ella
0.49
3.87
milliVolts
NL: 0
TIC F: + c ESI Full ms [150.00-1000.00] MS JS-Ella
Relative Abundance
Time (min)
NL: 2.37E7
JS-Ella#266 RT: 4.07
AV: 1 T: + c APCI corona Full ms [150.00-1000.00]
652.23
249.52
309.20
377.16
499.90
610.31
668.09
819.23
929.84
NL: 2.18E4
JS-Ella#207 RT: 3.20
AV: 1 T: + c APCI corona Full ms [150.00-1000.00]
194.03
652.19
283.97
479.12
686.19
NL: 7.77E4
JS-Ella#226 RT: 3.49
AV: 1 T: + c APCI corona Full ms [150.00-1000.00]
666.05
263.29
246.38
323.15
379.16
480.59
652.35
678.67
688.20
853.46
954.87
m/z

Starting material; corresponds to lit. data

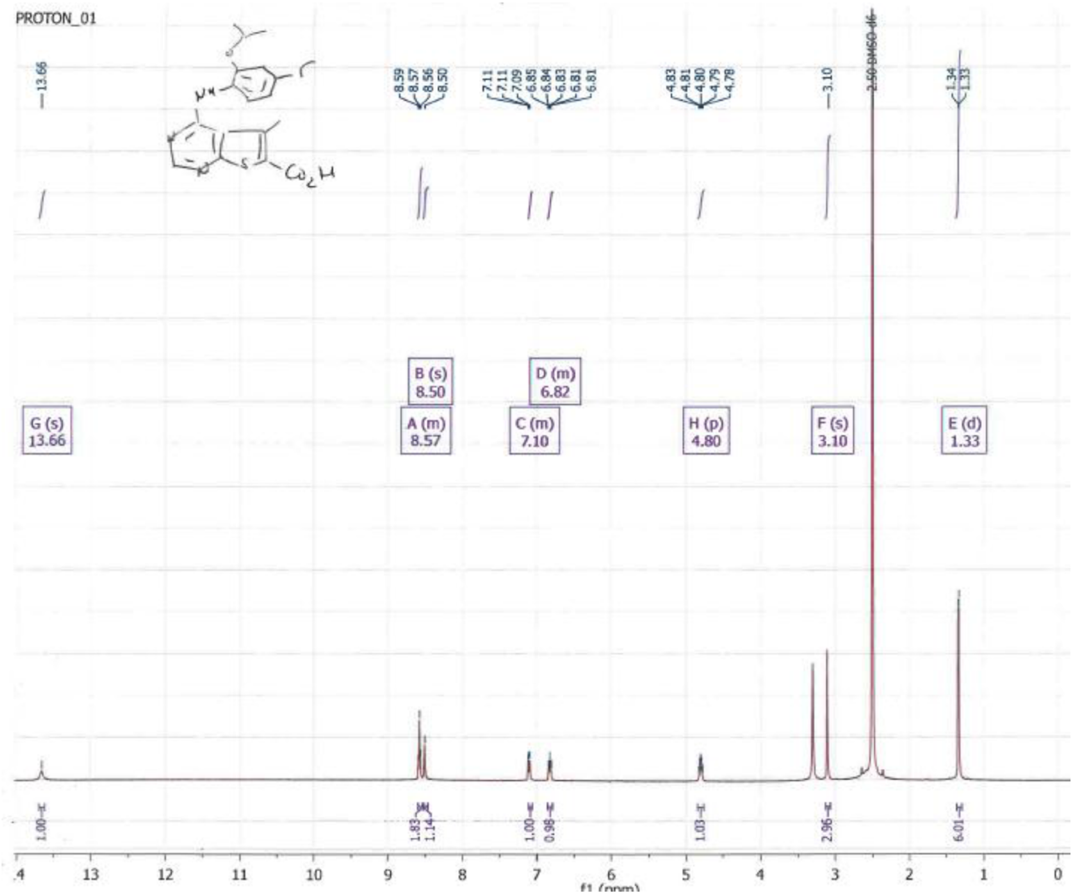

CHN analysis:

SAMPLE	%C	%H	%N
ella mnk	56.35 56.39	4.32 4.38	11.54 11.56

Stephen Boyer
Science Centre
London Metropolitan University
29 Hornsey Road
London N7 7DD

Supplementary Figure 1: ^{1}H NMR (DMSO-d6) of starting material 1: 4-((4-fluoro-2-isopropoxyphenyl)amino)-5-methylthieno[2,3-d]pyrimidine-6-carboxylic acid.

Supplementary Table 1: Physiochemical properties and lipophilicity prediction of inhibitors used in this study, calculated using SwissADME software [1]

Physiochemical Properties	MNKI-19	191.3	PP242	1	2
Molecular weight	361 .39 g/mol	313.37 g/mol	308.34 g/mol	656.75 g/mol	651.71 g/mol
Rotatable bonds	5	2	2	9	9
H-bond acceptors	6	4	4	9	9
H-bond donors	2	1	3	1	3
TPSA (Topological Polar Surface Area)	112.58 Å^2	86.72 Å^2	105.64 Å^2	168.07 Å^2	186.99 Å^2
Lipophilicity					
Log $P_{0/w}$	3.67	2.61	1.98	6.35	5.72
Druglikeness					
Lipinski	Yes	Yes	Yes	2 violations: MW > 500 Log P > 5	2 violations: MW > 500 Log PLog P > 5
Veber	Yes	Yes	Yes	1 violation: TPSA > 140	1 violation: TPSA >140

SUPPLEMENTARY REFERENCE

1. Daina A, Michielin O, Zoete V. SwissADME: a free web tool to evaluate pharmacokinetics, drug-likeness and medicinal chemistry friendliness of small molecules. Sci Rep. 2017; 7:42717.
